# Complete heart block and itchy rash in a patient with COVID-19

**DOI:** 10.22088/cjim.11.0.569

**Published:** 2020

**Authors:** Mohammad Dehghani Firouzabadi, Sogand Goudarzi, Fatemeh Dehghani Firouzabadi, Fatemeh Moosaie

**Affiliations:** 1 Endocrinology and Metabolism Research Center (EMRC), Vali-Asr Hospital, School of Medicine, Tehran University of Medical Sciences, Tehran, Iran.; 2 ENT and Head and Neck Research Center and Department, Five Senses Health Research Institute, Hazrat Rasoul Akram Hospital, Iran University of Medical Sciences, Tehran, Iran; 3 Division of Cardiovascular Medicine, Department of Medicine, Beth Israel Deaconess Medical Center, Harvard Medical School, Boston, MA, United States; # These authors contributed equally to this work

**Keywords:** Arrhythmia, Complete heart block, SARS-CoV-2, COVID-19, Skin Manifestation, Cutaneous Rash, Itching rash

## Abstract

**Background:**

The outbreak of coronavirus disease 2019 (COVID-19) has become a global crisis, as the World Health Organization (WHO) declared COVID-19 as a global pandemic. Complete heart block, resulting from an abnormal heart rhythm, is a rare presentation of this infection, which can be life-threatening due to possible progression into ventricular tachycardia.

**Case Presentation:**

We report a critical case of COVID-19 in a young woman without any medical history. She was admitted to the hospital with a rare, but serious presentation of temporary complete heart block with a skin rash after three weeks of treatment with an antiviral agent and hydroxychloroquine. The result of cardiac monitoring, using a Holter monitor, was normal, and her sinus rhythm returned to normal without any interventions.

**Conclusion:**

This case emphasized the importance of regular follow-ups for patients with COVID-19 and highlighted the need for attention to unusual presentations, such as brief episodes of unconsciousness and chest pain.

A novel coronavirus (SARS-CoV-2) is responsible for the outbreak of a disease, known as coronavirus disease 2019 (COVID-19). The presentations of COVID-19 can vary, ranging from asymptomatic cases to patients with multiple organ dysfunction syndromes, requiring intensive care support and invasive mechanical ventilation (1, 2). Iran is among the first countries, struggling with the COVID-19 epidemic up to March 15, 2020 (3). The full spectrum of COVID-19 severity is not yet characterized. However, a report indicated a complete heart block in a 54-year-old man with COVID-19 infection(4) . Here, we present the second case of temporary atrioventricular block in a patient with COVID-19.

## Case presentation

A 38-year-old female nurse presented with high fever (>40°C), chills, dry cough, nausea, vomiting, loss of taste and smell, and myalgia during the outbreak of COVID-19. After the initial assessment, it was clear that her high fever, myalgia, and dry cough had continued for ten days. After the infectious disease specialists confirmed the diagnosis of COVID-19 via reverse transcription-polymerase chain reaction (RT-PCR) assay, she was administered hydroxychloroquine (200 mg, PO q12h) and oseltamivir (75 mg, PO q12h) for five days. Two days later, anorexia and diarrhea were added to her symptoms. Six days later, she also reported the loss of taste and smell and hair loss, which continued for ten days. No noticeable sign of abnormality in sensory or motor nerve function was observed during two weeks. Also, laboratory tests, including complete blood count, C-reactive protein, and erythrocyte sedimentation rate, were normal in her first admission.

**Figure 1:**
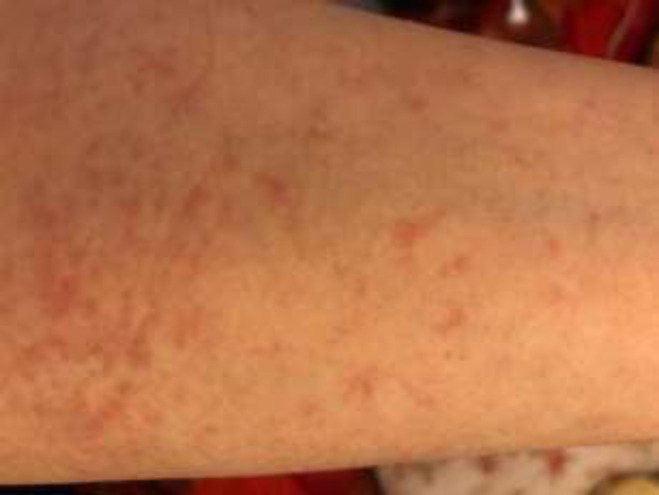
Itching skin rash on patient’s legs

After 20 days, the patient’s symptoms were completely relieved. However, she was admitted to the emergency room due to brief episodes of unconsciousness and chest pain at night, caused by an extremely sudden drop in pulse rate, as reported by her husband at home and coworkers during the shifts. In the emergency room, she had no fever (36.9°C), and her respiratory rate and oxygen saturation level were normal. The skin examination showed a pruritic rash, distributed on her breasts, shoulders, and legs, which were associated with her fainting episodes (figure 1). 

**Figure 2:**
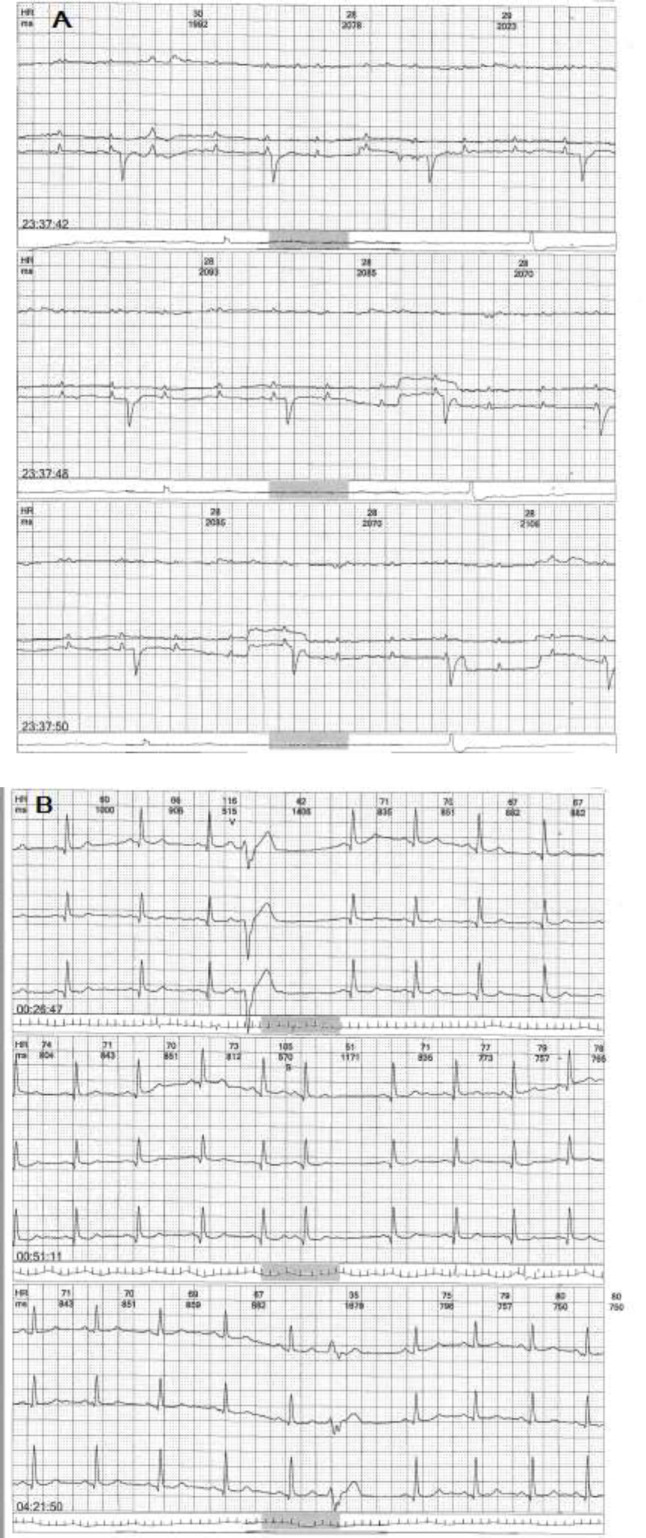
Holter monitoring test. A; First 24-hour Holter monitoring test indicating an atrioventricular block. B; Second 24-hour Holter monitoring test showing normal sinus rhythm

Cardiac monitoring was performed during one of her shifts when she fainted. The results showed bradycardia, and premature ventricular contractions (PVC) taken within less than 15 minutes. Next, 24-hour Holter monitoring was performed, which showed a temporary complete heart block (figure 2A). Laboratory tests, including complete blood count, troponin, creatine kinase-MB, D-dimer, C-reactive protein, erythrocyte sedimentation rate, lactate dehydrogenase, and COVID-19 IgM antibody rapid test were completely normal during her hospitalization. She was retested with PCR assay for COVID-19, and the result was normal in the third day of her second hospitalization.

She did not show any defects on her chest CT scan during her first and second hospitalizations for COVID-19. The cardiologists decided to use a pacemaker for her. After four days of assessment, the result of Holter monitoring was normal, and her sinus rhythms and PVCs returned to normal without any interventions (figure 2B). Accordingly, she was discharged from the hospital. Echocardiography was normal, with an ejection fraction up to 60% without pericardial effusion. Also, her skin rash disappeared one day after admission. After two weeks, she did not have any symptoms, her cardiac monitoring was normal, and all of her symptoms were completely relieved.

## Discussion

COVID-19 may lead to myocardial damage and arrhythmic or cardiovascular complications due to inflammatory responses, regardless of the patient’s medical history or RT-PCR results (depending on the gap between the presentation of initial COVID-19 symptoms and heart failure) (5, 6). In COVID-19 patients with bradyarrhythmia, complete heart block is considered as one the most common occurrences (7). In this regard, Azarkish et al. reported a case of complete heart block secondary to COVID-19, followed by tachypnea after 13 days of admission to a hospital (4). In another report, a young child, infected with SARS-CoV-2, presented with a complete heart block, which was suspected to result from high inflammatory responses (8). Another possible reason for the complete heart block in COVID-19 patients is the use of hydroxychloroquine, which can lead to the prolongation of QT intervals. Also, another side effect of hydroxychloroquine administration may be fascicular block, and consequently, advanced atrioventricular block and syncope (5).

Here, we reported a case of COVID-19 with complete heart block. This complication is likely to occur, even if the patient with COVID-19 has recovered. Our young patient, despite having no medical history and normal laboratory results after the second hospitalization, showed a sudden complete heart block. Overall, cardiac involvement can be a poor prognostic factor for COVID-19, which is difficult to detect in early stages and requires swift attention. Therefore, further global clinical studies with longer follow-ups are needed to determine if SARS-CoV-2 can cause permanent cardiac damage.
